# Inclusion criteria update for the rat intraluminal ischaemic model for preclinical studies

**DOI:** 10.1242/dmm.029868

**Published:** 2017-12-01

**Authors:** Héctor Fernández-Susavila, Ramón Iglesias-Rey, Antonio Dopico-López, María Pérez-Mato, Tomás Sobrino, José Castillo, Francisco Campos

**Affiliations:** Clinical Neurosciences Research Laboratory, University Clinical Hospital of Santiago de Compostela, Health Research Institute of Santiago de Compostela (IDIS), 15706 Santiago de Compostela, Spain

**Keywords:** Animal model, Cerebral ischaemia, Inclusion criteria, Laser Doppler, MR angiography, MRI

## Abstract

Proper occlusion of the medial cerebral artery, as determined by laser Doppler monitoring, during cerebral ischaemia in rat models is an important inclusion criterion in experimental studies. However, successful occlusion of the artery does not always guarantee a reproducible infarct volume, which is crucial for validating the efficacy of new protective drugs. In a rat intraluminal ischaemic model, laser Doppler monitoring alone was compared with laser Doppler monitoring in combination with magnetic resonance angiography (MRA) and diffusion-weighted imaging (DWI). Twenty-eight animals showed successful occlusion and reperfusion determined with Doppler monitoring, with an infarct size at 24 h of 16.7±11.5% (determined as ischaemic damage with respect to the ipsilateral hemisphere volume). However, when arterial occlusion and infarct damage were analysed in these animals using MRA and DWI, respectively, 15 animals were excluded and only 13 animals were included, with an infarct size at 24 h of 21.6±6.1%, showing a variability in the infarct size significantly lower (*P*<0.05, *F*-test) than that obtained with Doppler monitoring alone. We also observed that blocking of the pterygopalatine artery (a maxillary artery that is usually occluded in the intraluminal ischaemic model) was not relevant for this model, at least in terms of infarct variability. These results show that laser Doppler monitoring is a necessary procedure, but not sufficient to guarantee a reproducible infarct volume, in a rat ischaemic model. Therefore, laser Doppler monitoring in combination with DWI and MRA represents a reliable inclusion protocol during ischaemic surgery for the analysis of new protective drugs.

## Abstract (precis)

**Summary:** Laser Doppler monitoring in combination with diffusion-weighted imaging and magnetic resonance angiography represents a reliable inclusion protocol during ischaemic surgery for the analysis of protective drugs in the acute phase of stroke.

## INTRODUCTION

The Stroke Therapy Academic Industry Roundtable (STAIR) criteria have been updated periodically since their creation, with the purpose of improving the quality of preclinical studies on acute stroke therapies (Saver et al., 2013; Stroke Therapy Academic Industry Roundtable, 1991). One of the most crucial STAIR recommendations is the monitoring of cerebral blood flow (CBF) using laser Doppler during surgery to guarantee proper medial cerebral artery (MCA) occlusion (MCAo) and reproducible infarct size, usually determined at 24 h through magnetic resonance (MR) imaging (MRI) or histological techniques (Saver et al., 2013; Stroke Therapy Academic Industry Roundtable, 1991). In addition, in preclinical studies focusing on protective strategies for the acute phase of stroke (<12 h), Doppler flow monitoring represents the gold standard inclusion criterion used before treatment administration. Indeed, many researchers have demonstrated the efficacy of protective drugs based on Doppler flow monitoring, with all animals included in the study showing a reduction in CBF during a MCAo >70% or 80% from the basal levels. However, it is well known that the cerebral collateral circulation can supply blood to the ischaemic region that is difficult to register with the Doppler probe, and increases the internal variability of the experimental groups (Cuccione et al., 2016).

In this study, we intended to analyse, for the first time, the use of laser Doppler monitoring alone and in combination with diffusion-weighted imaging (DWI) and MR angiography (MRA) during MCAo, and to determine the infarct size variability at 24 h in both protocols.

## RESULTS

In this study, two different experimental inclusion protocols were compared: (1) inclusion of animals based on laser Doppler monitoring: animals with CBF reduction >70% and complete reperfusion (>60%) after MCAo determined only with laser Doppler monitoring; (2) inclusion of animals based on laser Doppler and MRI (DWI and MRA) monitoring during MCAo: animals with CBF reduction >70% determined with laser Doppler monitoring, DWI hemispheric infarct volume between 25% and 45% (indicated as the percentage of ischaemic damage with respect to the ipsilateral hemisphere volume), MRA of the MCAo, and complete reperfusion after MCAo. In both inclusion protocols, the relevance of occlusion of the pterygopalatine artery (PPA) was also tested.

**Fig. 1.**
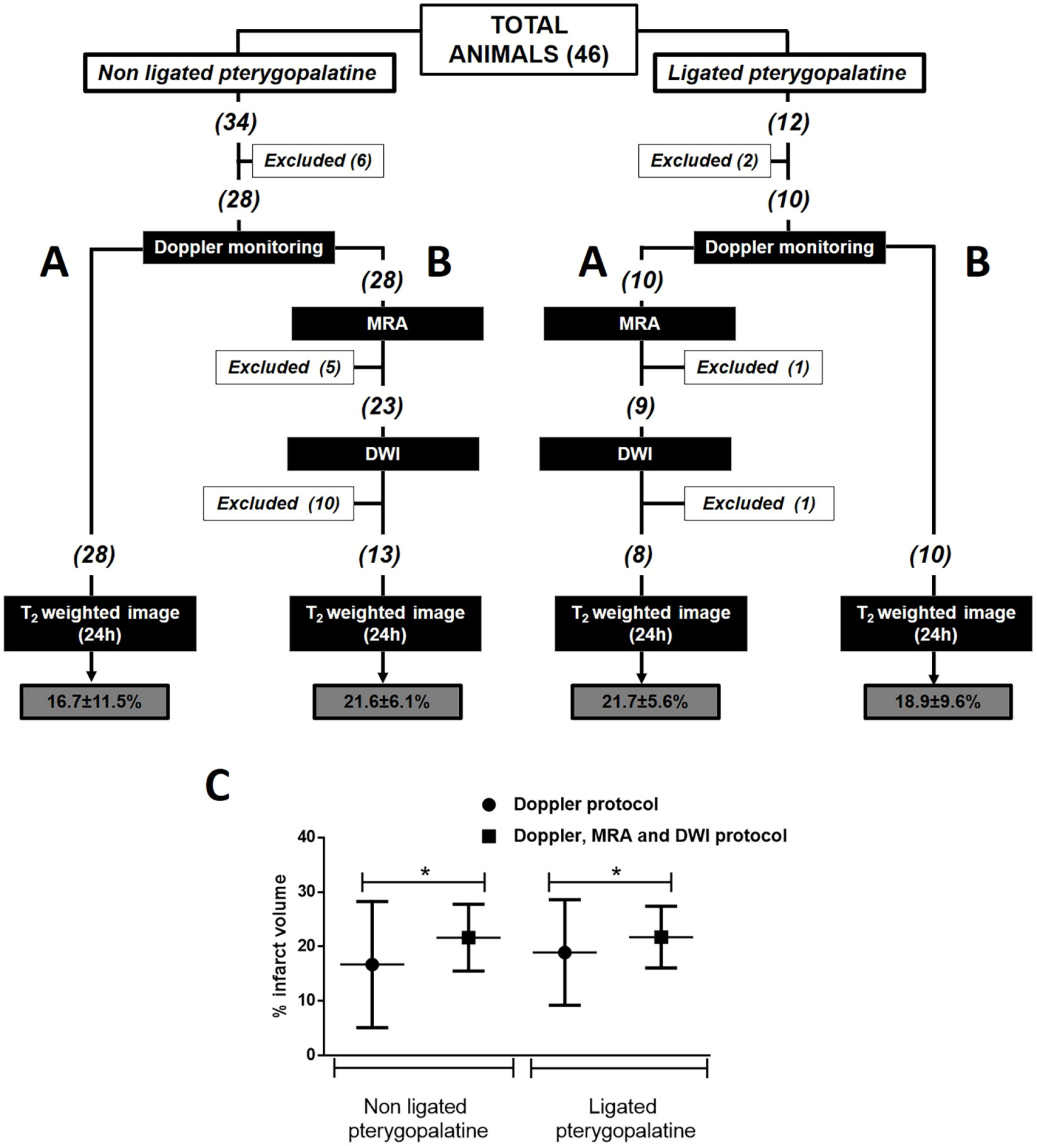
**Protocol diagram summarizing the number of animals included, with exclusions** **per group, for final analysis.** Two experimental groups were compared: (A) animals evaluated with only laser Doppler monitoring; (B) animals evaluated with laser Doppler monitoring, MR angiography and DWI. The two groups were compared in animals with and without occlusion of the pterygopalatine artery. (C) Infarct volume determined 24 h after ischaemia for the two inclusion protocols used in rats with and without pterygopalatine artery occlusion. Data are expressed as mean±s.d. Student's *t*-test was used to compare the differences between the means, and *F*-test was used to compare differences in variability. Means were similar for both inclusion protocols, while the variability was significantly reduced with the new inclusion protocol suggested (*P<0.05).

**Fig. 2.**
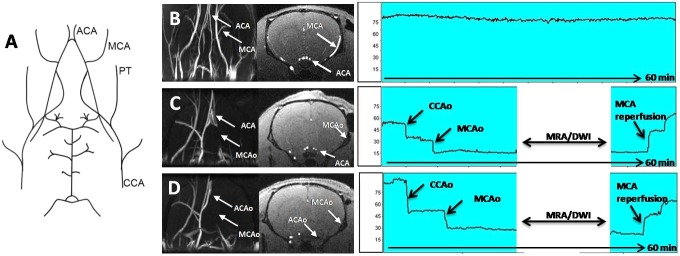
**The main aspects** **of** **the angiography technique.** (A) The cerebrovascular anatomy of the rat. (B) Coronal projection of a MR angiography image of a healthy rat. The ACA and MCA can be observed in the MR angiography projection (right) and in the axial image (middle). (C) Ischaemic animal with the MCA and the ACA occluded. (D) Ischaemic animal with only the MCA occluded. In the laser Doppler recording, animals with only the MCA occluded and animals with both the MCA and ACA occluded showed the same cerebral blood flow profile during arterial occlusion and reperfusion. ACA, anterior cerebral artery; ACAo, anterior cerebral artery occlusion; CCA, common carotid artery; CCAo, common carotid artery occlusion; MCA, middle cerebral artery; MCAo, middle cerebral artery occlusion; PT, pterygopalatine artery.

**Fig. 3.**
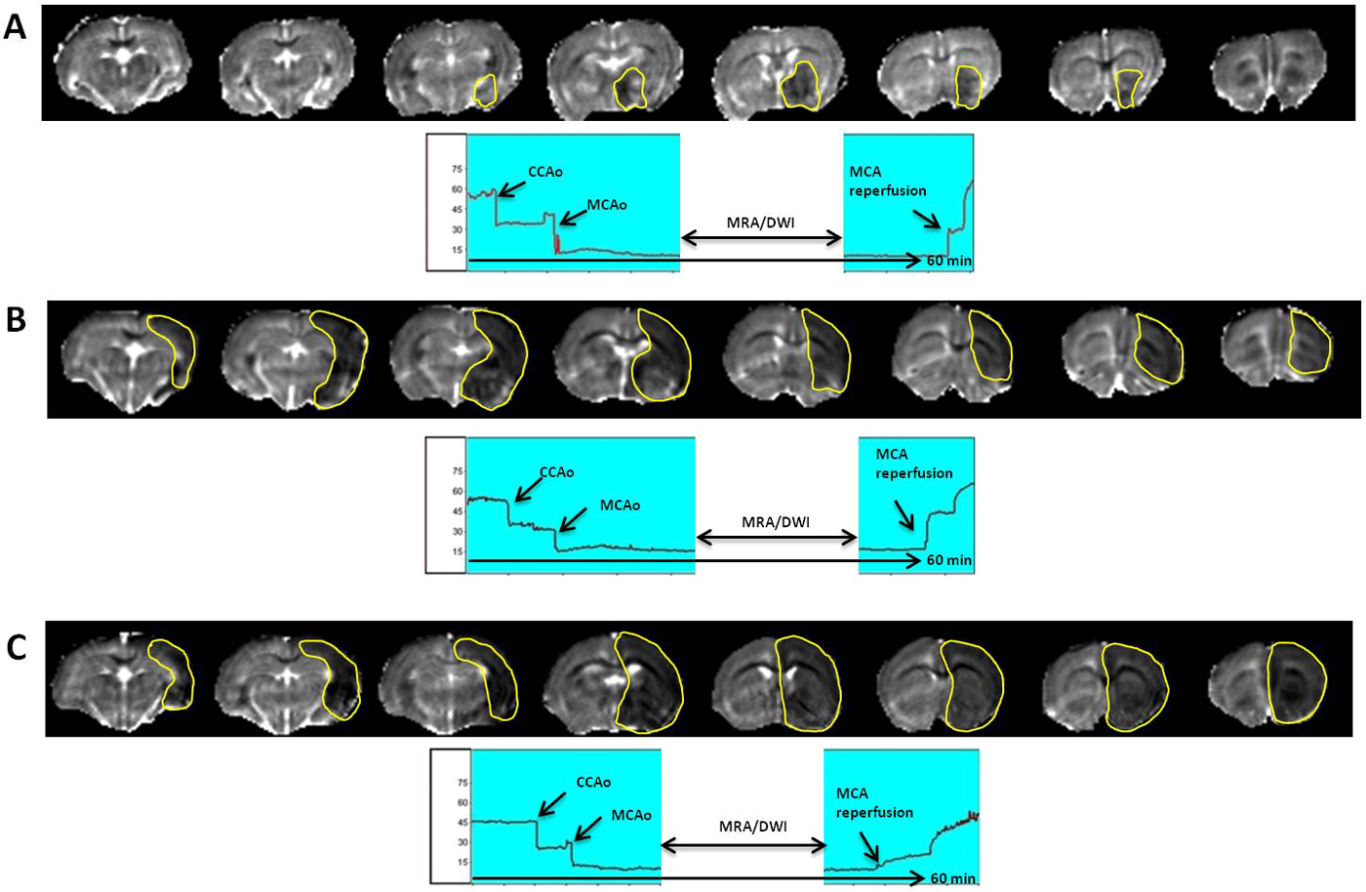
**Representative ADC maps (obtained from DWI) of animals included or excluded in the study.** (A) ADC maps of an animal excluded owing to a baseline lesion volume <25% of the ipsilateral hemisphere. DWI results, from which the ADC maps were obtained, are shown above. (B) ADC maps of an included animal with a baseline lesion volume within the accepted range (25-45% of the ipsilateral hemisphere). (C) ADC maps of an animal excluded owing to a baseline lesion volume >45% of the ipsilateral hemisphere. In the laser Doppler recording (below), animals with different DWI volumes presented the same cerebral blood flow profile during arterial occlusion and reperfusion.

A total of 34 animals were included (Fig. 1). Initially, six animals were excluded because of bleeding and spontaneous death during surgery. On the basis of Doppler monitoring, the remaining animals (*n*=28) had successful MCAo (>70% with respect to the basal level) and reperfusion (>60%), 60 min after occlusion. However, when these 28 animals were analysed by MRA during arterial occlusion, five were excluded because both the MCA and the anterior cerebral artery (ACA) had been occluded (Fig. 2). Moreover, when DWI was performed on the remaining 23 animals, 10 animals were excluded because the infarcted regions were out of the established range (25-45%) (Fig. 3). The DWI volume of these 13 finally included animals was 33.7±6.6%. Analysis of the ischaemic damage determined at 24 h showed that the infarct size in those animals included following the criterion of laser Doppler alone was 16.7±11.5% (variability 69%), whereas in those animals included based on the criterion of Doppler monitoring in combination with MRI analysis, the infarct size was 21.6±6.1% with a lower variability (28%) (*P*<0.05, *F*-test).

To validate whether PPA occlusion during ischaemic surgery could affect the previously observed infarct variability, the same procedure was performed. A total of 12 animals were initially included (Fig. 1). Two animals were excluded owing to complications during surgery and the other 10 passed the Doppler criteria. When the MRI protocol was performed on these animals, one animal was discarded in the angiographic analysis because the MCA and ACA were occluded, and one other animal was excluded because the infarct size was lower than the established DWI threshold. The average DWI volume in the included animals was 34.7±5.4% (*n*=8). The infarct size determined 24 h later in those animals that passed the Doppler criteria was 18.9±9.6% (variability 51%); however, in those animals subjected to both Doppler and MRI inclusion criteria, the infarct size was 21.7±5.6%, with a variability (26%) significantly lower (*P*<0.05, *F*-test).

The data obtained from this study also show that if we perform a power analysis to determine the number of animals per group required to predict a 30% reduction in lesion volume between the control group and a treated group, with this new inclusion protocol, fewer animals per group are needed (Table 1). Results are shown if we assume a power of 0.8, a significance level of 0.05, and predict a 30% reduction in lesion volume between the control group and treated group.

**Table 1.**
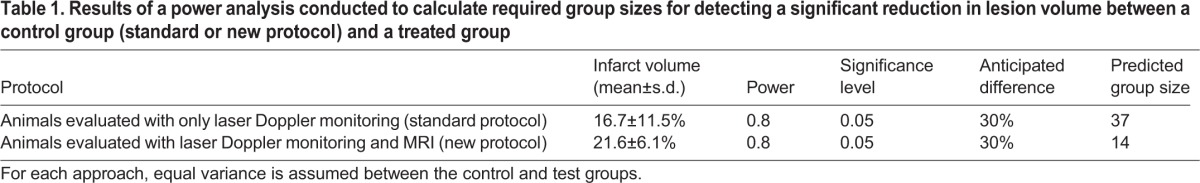
**Results of a power analysis conducted to calculate required group sizes for detecting a significant reduction in lesion volume between a control group (standard or new protocol) and a treated group**

## DISCUSSION

As shown by a recent publication about the limitations of translational stroke research (Dirnagl, 2016), an update of the inclusion criteria in ischaemic animal models, as described in this study, is needed to guarantee the efficacy of new protective drugs for the acute phase of stroke. Here, we propose a new inclusion protocol for the intraluminal ischaemic rat model, based on the combination of laser Doppler monitoring and MRI. This protocol also demonstrates that laser Doppler monitoring is needed to confirm MCAo during surgery; however, it does not guarantee a reproducible infarct size at 24 h, which is critical for the evaluation of drugs. Thus, animals with an identical arterial occlusion rate (<70% with respect to the basal flow) and similar recovery flow (>60%) after reperfusion, as determined by laser Doppler monitoring, presented a variability of 69%, whereas those selected on the basis of the combination of laser Doppler and MRI monitoring showed a variability of 28%.

Other studies have previously addressed the usefulness of laser Doppler for predicting infarct volume and outcome in MCAo models of stroke (Hedna et al., 2015; Reith et al., 1994; Taninishi et al., 2015). This study does not intend to suggest that laser Doppler monitoring is not a useful inclusion criterion. Indeed, laser Doppler is a useful tool for monitoring MCAo; however, because laser Doppler has a low sensitivity for measuring the collateral circulation (Cuccione et al., 2016), it cannot be used to exclude animals with extreme infarct sizes as DWI can. In addition, laser Doppler monitoring allows the detection of the MCAo when the filament reaches the circle of Willis. However, once the MCA is occluded, it becomes almost impossible with this technique to detect whether the ACA circulation is being altered, which is a crucial issue for obtaining reproducible ischaemic sizes. In this regard, angiographic imaging allows the exclusion of those ischaemic animals in which the MCA and ACA are occluded, reducing the variability of the infarct size at 24 h.

The PPA is a maxillary artery that supplies blood to the deep structures of the face and, in some surgical protocols for ischaemia induction, this artery is occluded to avoid the accidental intubation of this vessel with the intraluminal suture. However, this procedure can significantly influence the MCAo model and cannot be ignored (Cuccione et al., 2016). Therefore, this variable was also included in the experimental groups. In our analysis, we observed that PPA blocking during surgery is not relevant for this model, at least in terms of infarct variability.

It could be hypothesized that histological analysis of the ischaemic region is necessary to confirm the MRI measurement of infarct size 24 h after ischaemia. In this regard, it is now well established that MRI is the gold standard method for imaging and an effective *in vivo* alternative to histological evaluation for estimating treatment effects based on the extent of infarction (Milidonis et al., 2015). In addition, infarct analysis at 24 h was chosen because this represents one of the most common time points used in preclinical studies to validate the efficacy of protective treatments in the acute phase of stroke (O'Collins et al., 2011). However, we admit that the short time point used (24 h) and the lack of histological analysis are limitations of this study, as we were not able to detect haemorrhagic lesions, or to examine the long-term evolution of the ischaemic lesion and its impact on neurological functions.

Like all protocols, the use of MRI in combination with laser Doppler monitoring as an animal inclusion criterion in experimental ischaemic studies has disadvantages and advantages. Because the animal has to be moved after MCAo from the bench to the MR system (see Materials and Methods), one of the most important limitations in implementing this protocol is that the MRI facility must be close to the surgery bench to keep the animals under anaesthetized conditions, and to reduce the movement of the filament located in the artery as much as possible. This setup is not common in many research centres, and the situation becomes worse when the MRI facility is in a different building from the surgery facilities. We have also established an arbitrary inclusion interval (between 25% and 45%) for DWI hemispheric infarct volume that could be a topic for discussion. We established this threshold based on previous studies (Argibay et al., 2017; Campos et al., 2011; Pérez-Mato et al., 2014; Vieites-Prado et al., 2016) in which the same inclusion protocol was used, and in which DWI volumes <25% during MCAo were associated with small subcortical ischaemia or no ischaemia at 24 h, and DWI volumes >45% were associated with a malignant infarct that affected all hemispheres and resulted in a high mortality rate. Finally, this study was performed in Sprague-Dawley rats because this is the most common strain used in studies on cerebral ischaemia; however, we are aware that this protocol should be validated in other strains and species of rat, as well as in a permanent MCAo model. Despite these limitations, we would like to highlight that this new inclusion protocol allows reducing the infarct size variability 24 h after ischaemia, which is crucial for calculating required group sizes for detecting a significant reduction in lesion volume between a control group and a tested group. In fact, using the data obtained in this study, our power analysis shows that fewer animals per group are required to demonstrate a 30% reduction in lesion volume following this new protocol compared with the traditional one. In addition, this protocol also permits determining basal ischaemic lesions in the included animals before treatment administration, which, in combination with the ischaemic size determined after treatment, enhances the quality and reliability of the results.

In brief, we can conclude that laser Doppler monitoring is a necessary procedure, but not sufficient to guarantee a reproducible infarct volume, in the rat ischaemic model. Laser Doppler monitoring in combination with DWI and MRA represents a reliable inclusion protocol during ischaemic surgery for the analysis of new protective drugs focused on the acute phase of stroke.

## MATERIALS AND METHODS

### Animals

All experimental protocols were approved by the local Animal Care Committee according to the guidelines established by the European Union (86/609/CEE, 2003/65/CE, and 2010/63/EU) and following the Animal Research: Reporting of *In Vivo* Experiments (ARRIVE) guidelines for animal experiments. Male Sprague-Dawley rats weighing between 280 g and 330 g were used (aged 11-12 weeks). The animals were housed individually at an environmental temperature of 23°C, with 40% relative humidity and a 12 h light-dark cycle, and were given free access to food and water.

### Rat model of cerebral ischaemia and MRI

All surgical procedures were performed under sevoflurane anaesthesia (6% induction and 4% maintenance in a mixture of 70% NO_2_ and 30% O_2_). Rectal temperature was maintained at 37±0.5°C in all animals during surgery, by using a thermostat-controlled electric pad (Neos Biotec, Pamplona, Spain). The head of the animals was placed on a porexpan plate to avoid direct contact between the pad and the head. Transient focal ischaemia (60 min) was induced through intraluminal MCAo, following methods described previously (Howells et al., 2010; Lee et al., 2014). All surgeries were performed by one researcher (>2 years’ experience) in the transient intraluminal filament MCAo model.

Occlusion was performed using commercially available sutures with silicone-rubber-coated heads (350 µm diameter and 1.5 mm length; Doccol, Sharon, MA, USA). CBF was monitored with a Periflux 5000 laser Doppler perfusion monitor (Perimed AB, Järfälla, Sweden) by placing the Doppler probe (model 411, Perimed AB) under the temporal muscle at the parietal bone surface, near the sagittal crest. Twenty-five minutes after arterial occlusion had been achieved, as indicated by Doppler signal reduction (CBF reduction >70%), each animal was carefully and immediately (within <1 min) moved from the surgical bench to the MR system for ischaemic lesion assessment using DWI. In combination with DWI, MRA was performed to ensure that the artery remained occluded throughout the MR procedure and to confirm the occlusion of only the MCA. The animals were then returned to the surgical bench and the Doppler probe was repositioned. Reperfusion was performed 60 min after occlusion onset. Animals with no reperfusion or with prolonged reperfusion (>10 min until achievement of at least 60% of the baseline CBF) after filament removal were not included. Ischaemic damage was confirmed and determined 24 h after ischaemia by using MR T2-weighted images. The transient intraluminal filament MCAo model was used because it represents the most common model used in the field of stroke experiments (Cuccione et al., 2016).

### Experimental groups

Two different experimental inclusion protocols were compared: (1) inclusion of animals with CBF reduction >70% and complete reperfusion after MCAo determined only with laser Doppler monitoring; (2) inclusion of animals with CBF reduction >70% determined with laser Doppler monitoring, DWI hemispheric infarct volume between 25% and 45% (indicated as percentage of ischaemic damage with respect to the ipsilateral hemisphere volume), MRA of the MCAo, and complete reperfusion after MCAo.

Both inclusion protocols were also compared in animals with occluded PPA.

### MRI

All studies were conducted on a 9.4-T horizontal bore magnet (Bruker BioSpin, Ettlingen, Germany) with 440 mT/m gradients and a combination of a linear birdcage resonator (70 mm in diameter) for signal transmission and a 2×2 surface coil array for signal detection. MRI postprocessing was performed with ImageJ software (https://imagej.nih.gov/ij/).

Basal ischaemic lesion during MCAo was determined by counting pixels with apparent diffusion coefficient (ADC) values below a threshold in the ipsilateral brain hemisphere. The values of ADC in the healthy rat brain normally do not fall below 0.55×10^−3^ mm^2^/s; therefore, this threshold provides a convenient means of segmenting abnormal tissue (Reith et al., 1995). ADC maps were obtained from diffusion-weighted images by using a spin echo echo-planar imaging sequence with the following acquisition parameters: echo time (TE)=26.91 ms; repetition time (TR)=4 s; spectral bandwidth (SW)=200 kHz; 7 b-values of 0, 300, 600, 900, 1200, 1600 and 2000 s/mm^2^; flip angle (FA)=90°; number of averages (NA)=4; 14 consecutive slices of 1 mm; field of view (FOV)=24×16 mm^2^ (with saturation bands to suppress signals outside this FOV); matrix size=96×64 (isotropic in-plane resolution of 250 μm/pixel×250 μm/pixel); and with fat suppression option.

To evaluate the status of MCAo in a noninvasive manner, time-of-flight (TOF) MRA was performed. The TOF-MRA scan was performed with a 3D-Flash sequence with TE=2.5 ms, TR=15 ms, FA=20°, NA=2, SW=98 kHz, 1 slice of 14 mm, FOV=30.72×30.72×14 mm^3^ (with saturation bands to suppress signals outside this FOV), matrix size=256×256×58 (resolution of 120 μm/pixel×120 μm/pixel×241 μm/pixel), and implemented without fat suppression option. DWI and TOF-MRA images were simultaneously acquired during MCAo (30±5 min after occlusion).

Ischaemic lesions were determined 24 h after ischaemia from T2 maps. These maps were calculated from T2-weighted images by using a multi-slice multi-echo sequence with TE=9 ms, TR=3 s, 16 echoes with 9 ms echo spacing, FA=180°, NA=2, SW=75 kHz, 14 slices of 1 mm, FOV=19.2×19.2 mm^2^ (with saturation bands to suppress signals outside this FOV), matrix size=192×192 (isotropic in-plane resolution of 100 μm/pixel×100 μm/pixel), and implemented without fat suppression option. Infarct size was indicated as the percentage of ischaemic damage with respect to the ipsilateral hemisphere volume, corrected for brain oedema. Image evaluations were performed by a researcher blinded to the experimental conditions.

### Statistical analysis

All data are expressed as mean±s.d. The data were analysed using GraphPad Prism v.6.05 for Windows (GraphPad). The criterion for statistical significance was *P*<0.05. Data were first examined to assess distribution using the D'Agostino and Pearson omnibus normality test. Parametric data comparing two means (MRI T2 scan) were compared using Student's *t*-test. Variability of data was assessed using the *F*-test for parametric data (MRI T2 scan). Sample size was calculated using EPIDAT software (http://www.sergas.es/Saude-publica/EPIDAT-4-2).
